# Area V1 responses to illusory corner-folds in Vasarely’s nested squares and the Alternating Brightness Star illusions

**DOI:** 10.1371/journal.pone.0210941

**Published:** 2019-03-28

**Authors:** Susana Martinez-Conde, Michael B. McCamy, Xoana G. Troncoso, Jorge Otero-Millan, Stephen L. Macknik

**Affiliations:** 1 Barrow Neurological Institute, Phoenix, AZ, United States of America; 2 State University of New York (SUNY) Downstate Medical Center, Brooklyn, NY, United States of America; 3 UNIC-CNRS (Unité de Neuroscience Information et Complexité, Centre National de la Recherche Scientifique), Gif-sur-Yvette, France; 4 Department of Neurology, Johns Hopkins University, Baltimore, MD, United States of America; Tokai University, JAPAN

## Abstract

Vasarely’s nested squares illusion shows that the corners of concentric squares, arranged in a gradient of increasing or decreasing luminance, generate illusory “corner-folds,” which appear more salient (either brighter or darker) than the adjacent flat (non- corner) regions of each individual square. The Alternating Brightness Star (ABS) illusion, based on Vasarely’s classic nested squares, further shows that the strength of these corner-folds depends on corner angle. Previous psychophysical studies showed the relationship between corner angle and perceived contrast in the ABS illusion to be linear, with sharp angles looking higher in contrast, and shallow angles lower in contrast. Center-surround difference-of-Gaussians (DOG) modeling did not replicate this linear relationship, however, suggesting that a full neural explanation of the nested squares and ABS illusions might be found in the visual cortex, rather than at subcortical stages. Here we recorded the responses from single area V1 neurons in the awake primate, during the presentation of visual stimuli containing illusory corner-folds of various angles. Our results showed stronger neural responses for illusory corner-folds made from sharper than from shallower corners, consistent with predictions from the previous psychophysical work. The relationship between corner angle and strength of the neuronal responses, albeit parametric, was apparently non-linear. This finding was in line with the previous DOG data, but not with the psychophysical data. Our combined results suggest that, whereas corner-fold illusions likely originate from center-surround retinogeniculate processes, their complete neural explanation may be found in extrastriate visual cortical areas.

## Introduction

**Fig 1:**
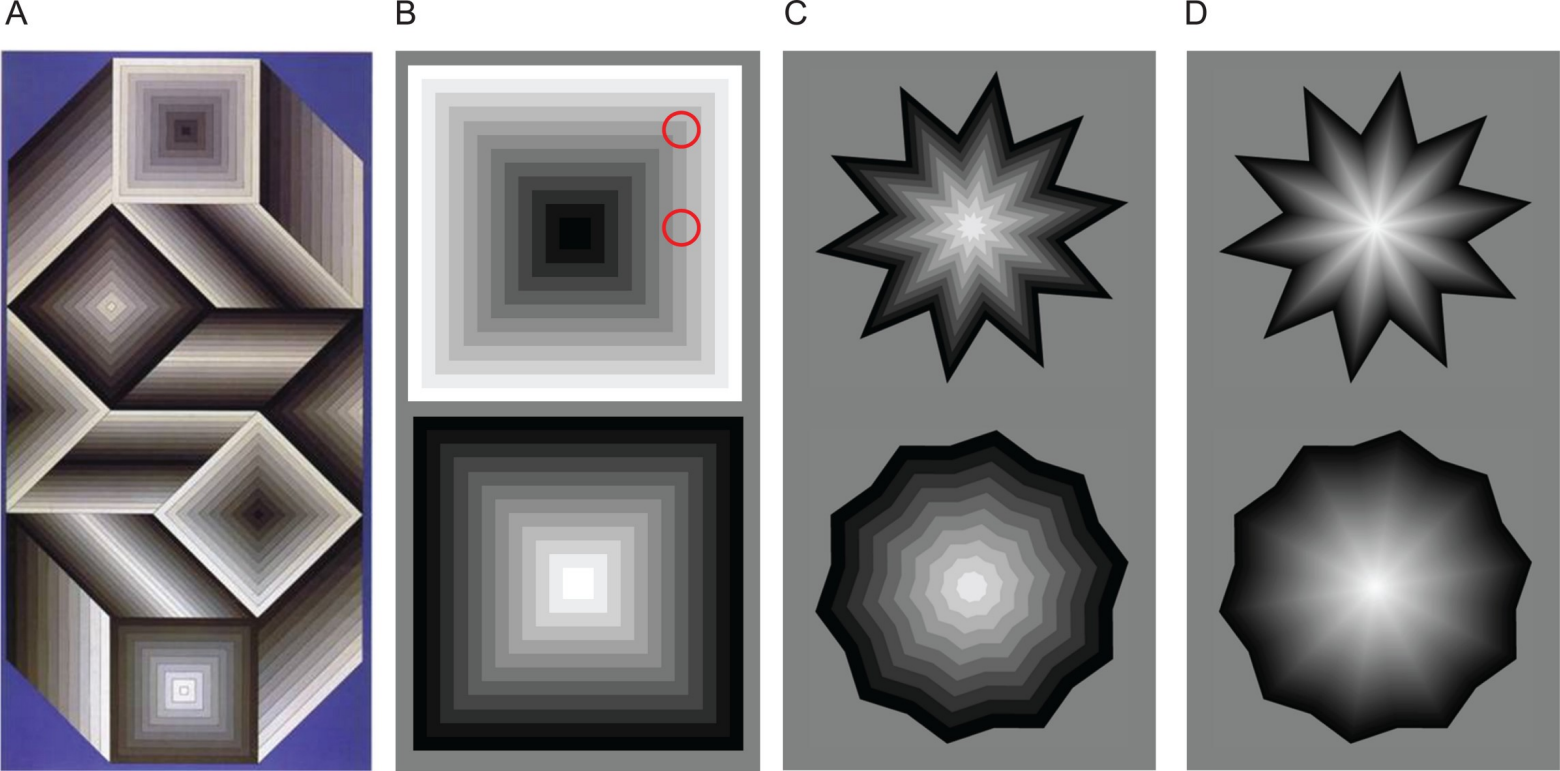
Vasarely’s nested-square and alternating brightness stars (ABS) illusions. Vasarely’s nested squares and ABS illusions. (**A**) Vasarely’s “Utem” (153 x 298 cm, 1981) [7]. Note the four sets of nested squares. The two nested squares of decreasing luminance (from the center to the outside) have bright illusory diagonals, whereas the two nested squares of increasing luminance (from the center to the outside) have dark illusory diagonals. The physical luminance of each individual square remains constant at all points, but the squares’ corners appear perceptually more salient than their straight edges, forming illusory X shapes that seem to irradiate from the very center of each set of squares. (**B**) Nested squares illusion based in Vasarely’s “Arcturus” [1]. Top: The stimulus is made out of multiple concentric squares of increasing luminance (from the center to the outside). The two red circles indicate two regions that seem to differ in brightness. The area inside the upper red circle has higher average luminance—but appears perceptually darker—than the region inside the lower circle. Bottom: Nested squares decreasing in luminance from the center to the outside. (**C, D**): The ABS illusion [2]. The stimulus is made of concentric stars of graded luminance. In the examples illustrated, the innermost star is white and the outermost star is black. The illusory corner-folds that radiate from the center appear as light or dark depending on the polarity of the corner angle (convex or concave). All illusory folds are physically equal to each other in luminance, but appear more salient with sharp corners (top stars) than with shallow corners (bottom stars). In (C) the gradient from the center to the outside has 10 luminance steps, making the individual stars forming the polygonal constructs easy to identify. In (D) the gradient from the center to the outside has 100 luminance steps. From [4].

Vasarely’s ‘nested squares’ illusion shows that the corners of concentric squares, arranged in a gradient of increasing or decreasing luminance, generate illusory diagonals, or “folds,” which appear more salient (i.e. brighter or darker) than the adjacent flat (non-corner) regions of each individual square (**Fig 1A and 1B**) [1]. The Alternating Brightness Star (ABS) illusion, which we developed based on Vasarely’s classic nested squares [2], further shows that the strength of the illusion depends on the corner angle, with sharp angles generating “corner-folds” that look higher in contrast, and shallow angles generating “corner-folds” that look lower in contrast (**Fig 1C and 1D**) (see http://smc.neuralcorrelate.com/demos/ABS-illusion.html for an interactive demonstration of the ABS illusion). Previous work attributed the nested squares and ABS illusions to changes in local contrast, and moreover found that applying a DOG (difference-of-Gaussians) filter to nested corners of varying angles resulted in outputs that qualitatively matched one’s subjective perception of the corresponding illusory folds [3]. Yet, psychophysical quantification of illusory strength revealed not just a parametric, but a *linear* relationship between corner angle and perceived contrast, which was not apparent in the DOG model’s output [3,4]. The mismatch between the DOG simulations and the psychophysical data suggested that a subcortical (i.e. center-surround) explanation of the nested squares and ABS illusions may be an incomplete one. However, no research to date has recorded the responses of visual system neurons—cortical or subcortical—during the presentation of these illusions.

Here we set out to measure the responses from neurons in the primary visual cortex to ABS-based illusions. To do this, we presented various types of images containing illusory corner-folds to awake rhesus monkeys, while we recorded the activity from single neurons in area V1. In agreement with previous psychophysical and DOG data, we found that sharper corners resulted in stronger neural responses, and shallower corners in weaker neural responses. We moreover found a parametric, but apparently non-linear, relationship between corner angle and strength of neuronal response: this result was consistent with the previous DOG data, but not with the psychophysical data. Our combined findings suggest that a) there may be little transformation of information from subcortical to primary visual cortical levels, in terms of the neural bases of corner-fold illusions, b) the previously found linear relationship between corner angle and subjective perception of illusory strength likely arises beyond area V1, and c) whereas corner-fold illusions are likely to originate from center-surround retinogeniculate processes, their complete neural explanation may only be found in areas of the extrastriate visual cortex.

## Materials and methods

### Surgical and recording procedures

We recorded single-neuron responses from area V1 of two adult rhesus monkeys (*Macaca Mulatta*, one male and one female), during the presentation of brightness illusions based on Vasarely’s ‘nested squares.’

The details of animal surgery, electrophysiological recordings, and receptive-field (RF) mapping have been reported previously in detail [5]. Briefly, each monkey was implanted with a head stabilization post, a scleral eye coil to monitor eye movements (sutured to the sclera to avoid slippage), and a recording chamber mounted over the occipital operculum to gain access to area V1. Implantation surgeries were conducted under general anesthesia using aseptic techniques, and with full post-operative analgesia and antibiotic therapy.

Surgical anesthesia was induced with ketamine (10 mg/kg IM). Gas inhalation anesthesia (0.5–1.5% Isoflurane) was administered throughout the implantation procedure. Respiration, pulse rate, CO2 levels and temperature were continuously monitored and recorded with the values of the anesthesia infusion rate and physiological monitoring (electrocardiogram, heart rate, oximetry, indirect arterial pressure, pupil size, withdrawal reflex, corneal reflex). Buprenorphine (0.005 mg/kg IM) was used for postoperative analgesia.

No animals were sacrificed at the end of the experiments, but were returned to Harvard Medical School’s primate colony. We followed the ARRIVE (Animal Research: Reporting of In Vivo Experiments) guidelines, and the Harvard Medical Area Standing Committee on Animals approved all surgical and electrophysiological methods. The ARRIVE Checklist is available as supporting information (S1 Checklist).

The animals were bred in captivity and housed individually in nonhuman primate cages (group 4; dimensions 89 cm width, 147 cm height, 125 cm depth, including perch) for the duration of the experiment. Monkeys were provided with several kinds of environmental enrichment, including various fruits and vegetables, food puzzles, perches, Kong toys, mirrors, and other enrichment tools as available, along with visual and auditory contact with several other monkeys that were also housed individually in the same room, and positive daily human contact. The room had a 12 hour light/dark cycle. Regular veterinary care and monitoring, balanced nutrition, and sensory and social environmental enrichment were provided in accordance to the National Institutes of Health Guide for the Care and Use of Laboratory Animals and the EU Directive 2010/63/EU for animal experiments, to maximize physical and psychological well-being. Monkeys had abundant access to food (i.e. feed biscuits were provided twice a day (approximately 12 biscuits/monkey), Purina Lab Diet Monkey Diet, Product # 0001333). Daily fluid intake was controlled and monitored during the experiments. Monkeys typically earned over 80% of their daily fluid allotment during the testing sessions, and received water and/or fruit supplements after the experiments. Whenever the animals were not actively participating in testing or training sessions (i.e. weekends, data analysis and manuscript writing periods, etc.), they had free access to water in the vivarium.

Each animal sat in a chair ~58 cm away from a CRT video monitor during the recordings. The size of the display was 40 degrees of visual angle (DVA) in width and 30 DVA in height. Screen resolution was set at 1024 × 768 pixels and the refresh rate at 60 Hz. Our in-house computer program (RF2) remapped the coordinates of the monkey display into 640 × 480 display units (DUs; i.e. 16 DUs per DVA).

We recorded eye movements using the search coil technique (C-N-C Engineering and Remmel Labs), at 1 kHz. Excursions of gaze outside of an invisible 2x2 DVA (32 × 32 DU) fixation window were recorded but not rewarded. We acquired extracellular single-unit potentials with lacquer-coated electropolished tungsten electrodes at 1 kHz. A time-amplitude-window discriminator (BAK Electronics, Inc.) was used to isolate the spikes on-line and only spike times were recorded. Each neuron’s receptive field (RF) was initially hand-mapped via computer-generated moving slits whose orientation, size, and rate of movement were manually controlled by the experimenter. Once we obtained an approximation of the RF size and location in this way, we then used a bar of optimum size and orientation that flashed in quick succession in various parts of the visual field, to more precisely determine the RF’s position and size [6]. The majority of neurons we recorded from were ON-dominant, and RF eccentricities ranged from 1–30 DVA.

### Visual stimuli

**Fig 2:**
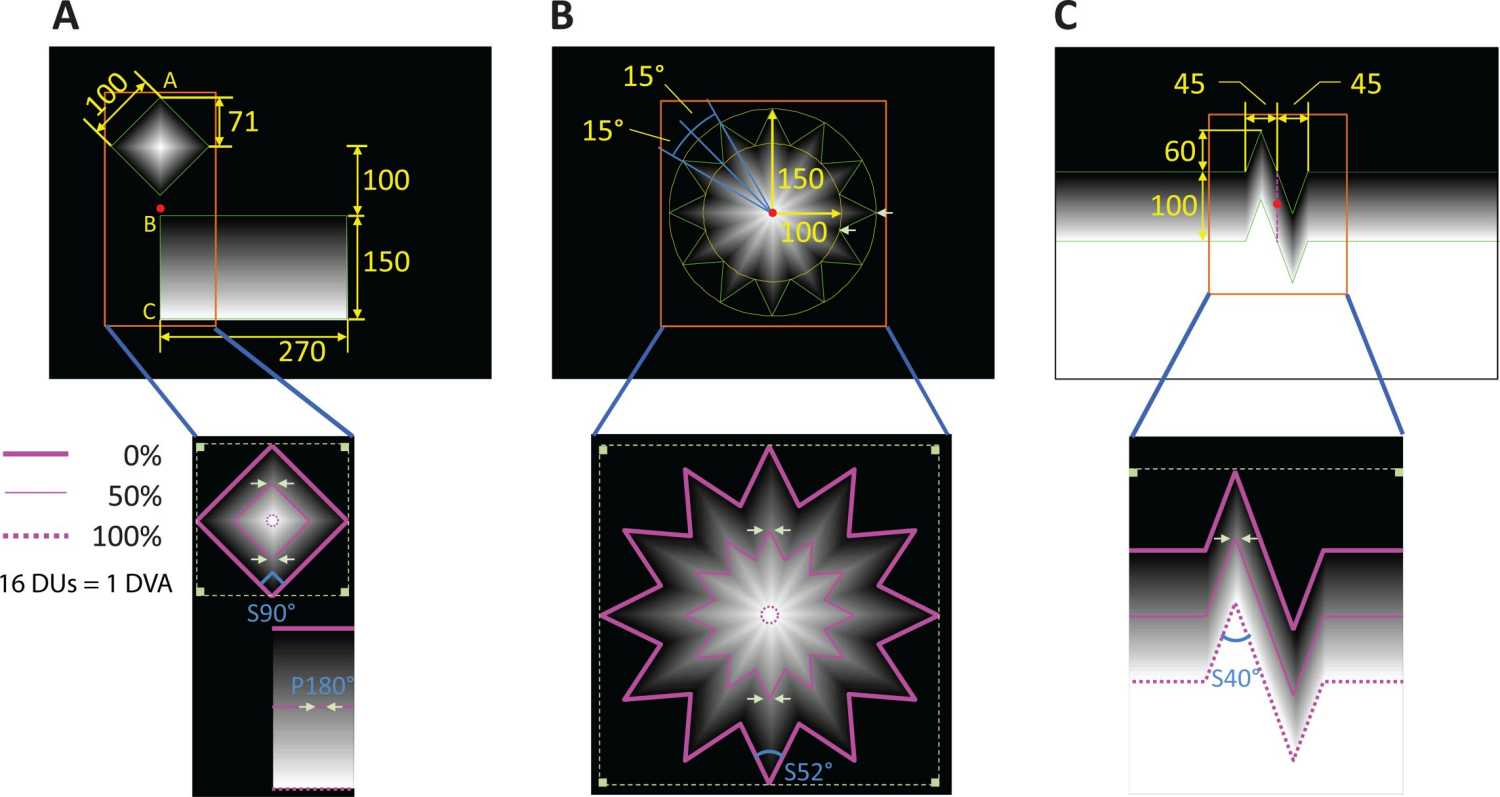
Visual stimuli. In each case, the upper panel shows the stimulus drawn to scale with the largest rectangle representing the extent of the monitor and the orange box indicating the ROI (the small red dots on the upper panels denote the center of each ROI) where neural responses were analyzed. The lower panels further specify each ROI, where the thick, thin, and dotted purple lines indicate the 0%, 50%, and 100% equal luminance lines. Each angle in the lower panels indicates that of a bright illusory fold (S90°, S52°, S40°) to which the neuron’s preferred orientation was aligned, or the neutral (i.e. flat) stimulus (P180°), where neural responses were further quantified. These responses were measured along the fold and also at the 50% luminance (i.e. the midpoint on the fold) locations in the 0.5x0.5 DVA ROIs denoted by the small white square frames flanked by white arrows. Finally, the larger white square frames in each of the lower panels indicate the 1x1 DVA ROIs where absolute baseline activity was measured (the physical squares within are 0% equal luminance squares). Any number without a degree symbol on it has units of DU. **(A)** Square and Edge. *Upper*: The green solid lines outline the Square and the rectangle displaying the luminance gradient. The line BC of the rectangle is the Edge. The midpoint of the line AC denotes the center of the ROI. *Lower*: Note that in the square the 100% equal luminance line contracts to a single point at the center, indicated with a small dotted circle. **(B)** Star. *Upper*: The green star polygon outlines the Star pattern. The Star is made of concentric, twelve-pointed, equilateral and equiangular star polygons, each polygon having homogeneous luminance throughout, and with the luminance of each successive polygon decreasing in value from the center outwards. The yellow outer and inner circles indicate the size of the outermost star (i.e. 0% luminance star). The two white arrows indicate one outer and one inner corner. The center of the ROI is at the Star’s center, indicated by the red dot. *Lower*: Note that in the Star the 100% equal luminance line contracts to a single point at the center, indicated with a small dotted circle. **(C)** Corners. *Upper*: The green lines outline the Corners pattern, which has rotational symmetry and includes two connected isosceles triangles (on the left and right of the purple dashed vertical line) with opposing directions. *Lower*: The Corners pattern forms four illusory folds, two with 40° angles, and the other two with 110° angles. We aligned the neuron’s preferred orientation to the 40° angle, so the 40° folds are symmetric (S40°), whereas the 110° folds are not. Refer to the **S1 Fig** for a clean display of all the stimuli drawn to scale.

We designed three types of visual stimuli displaying illusory folds formed by corners of various angles, as well as non-illusory stimuli of varying luminance/contrast: “Square and Edge”, “Star”, and “Corners” (**Fig 2**), which we describe next. Throughout the visual stimuli description, we give sizes in both DVA and DU, with 1 DVA corresponding to 16 DUs. We defined a “*corner-fold”* as an illusory fold whose bisector of fold angle is aligned with a neuron’s preferred orientation. Each individual stimulus had a region of interest (ROI) in which we quantified neuronal responses in (**Fig 2**–orange boxes), as described.

#### Square and edge

The Square and Edge stimulus (**Fig 2A**) consisted of a square (i.e. Vasarely’s nested-square) and an edge (the edge of a 16.88 × 9.38 DVA, or 270 × 150 DU, rectangular pattern comprised of linearly increasing luminance lines); heretofore, when referring to Square/Edge individually, we are referring to the Square/Edge component of Square and Edge. The square had a side length of 6.25 DVA (100 DU), with luminance decreasing from 100% to 0% at 22.53% per DVA from the center to the outside. The edge was on the extended line of one of the diagonals of the square (line BC of the rectangle in **Fig 2A**, and collinear with A), subtending 9.38 DVA (150 DU) with luminance increasing from 0% to 100% at 10.67% per DVA. The direction of the edge and the corresponding diagonal of the square were aligned with the neuron’s preferred orientation (i.e. direction of line AC in **Fig 2A**). The distance from the square center to the top of the edge was 6.25 DVA (100 DU). We measured the neural activity within the ROI (which measures 21.00 × 9.38 DVA) and we further quantified the following responses within it: 1) baseline responses to the Square and Edge, defined as the average activity in each of the four 1 × 1 DVA (16 × 16 DU) 0% luminance square areas at the corners of the 8.84 × 8.84 DVA (138 × 138 DU) circumscribing square (indicated by the gray dotted line in **Fig 2A**), 2) responses along the Edge, 3) responses along the 90° corner-fold (S90°), and 4) responses to the neutral (i.e. flat) fold that was 3 DVA (48 DU) away from Edge in the rectangle at the 50% luminance (14.07 cd per m^2^) line, denoted P180° (**Fig 2A**). Note, when we refer to folds anywhere in the text, P180° is included unless otherwise specified. Also, note that in some sessions the rectangle (but never its edge) was partially off the display, depending on the eccentricity of the neuron and the relative positions between the array (i.e. grid) of potential fixation positions and the stimulus (this had no effect on our analyses).

#### Star

The Star (**Fig 2B**) stimulus was formed with concentric star polygons, each of which was twelve-pointed, equilateral, and equiangular. The luminance of the concentric stars decreased from the innermost (100%) to the outermost (0%) polygon, at 10.76% per DVA along the diagonal. The radius of the circumscribed circle of the 0% equal luminance polygon was 9.38 DVA (150 DU) and the radius of the inscribed circle of the 0% equal luminance polygon was 6.25 DVA (100 DU) (see circles in **Fig 2B**). One of the diagonals connecting two opposite vertices of the 0% luminance star polygon was aligned with the neuron’s preferred orientation. We measured the neural activity within the ROI (which measured 10 × 10 DVA (160 × 160 DU)) and we further quantified the following neuronal responses within it: 1) baseline responses to the Star, defined as the average activity in each of the four 1 × 1 DVA (16 × 16 DU) 0% luminance square areas at the corners of the circumscribing square (indicated by the gray dotted line in **Fig 2B**), and 2) responses along the corner-fold of 52° (S52°) that was aligned with the neuron’s preferred orientation.

#### Corners

The Corners stimulus (**Fig 2C**) consisted of two isosceles triangles of identical dimensions, placed in opposite direction and connected successively. The base of each triangle was 2.81 DVA (45 DUs) and the height of each was 3.75 DVA (60 DUs). The angle of the apex of the isosceles triangle was ~40°. The luminance increased from 0% to 100% at 16% per DVA. The direction of the corner-fold of 40° (S40°) was aligned with the neuron’s preferred orientation. We determined the neural activity within the ROI (which measured 13 × 17 DVA (208 × 272 DU)) and further quantified the following neuronal responses within it: 1) baseline responses to Corners, defined as the average activity in two 1 × 1 DVA (16 × 16 DU) square regions in 0% luminance areas within the ROI (shown in **Fig 2C**), and 2) responses along the corner-fold S40° (also aligned with the neuron’s preferred orientation).

In **Fig 2**, for each of the three visual stimuli, the paired gray arrows in the lower panels indicate the positions where neuron’s midpoint (50% luminance) responses to the illusory bright fold were estimated. Note that Square has two symmetric midpoints along the diagonal. Thus, we first estimated neuronal responses at the two points and then averaged them to obtain the midpoint response to the square. Similarly, we first estimated the two midpoint responses of the neuron to the Star stimulus and then averaged them to obtain the midpoint response to the Star. Finally, we only analyzed symmetric bright corner-folds (i.e. bright folds aligned with the neuron’s preferred orientation) and P180° (which is the limit of a symmetric corner-fold). We did not analyze dark corner-folds (due to the relative scarcity of neurons with a preference for dark vs. bright stimuli in our sample) or non-symmetric corner-folds (the latter ones were only present in the Corners stimulus, where the 110° fold was not aligned with the neuron’s preferred orientation (meaning the preferred orientation did not bisect the fold angle)).

### Guided-viewing task

Once we identified each neuron’s preferred orientation and aligned a portion of the visual stimulus with it (see the Visual Stimuli section for details), the monkey performed the guided-viewing task we describe next.

**Fig 3:**
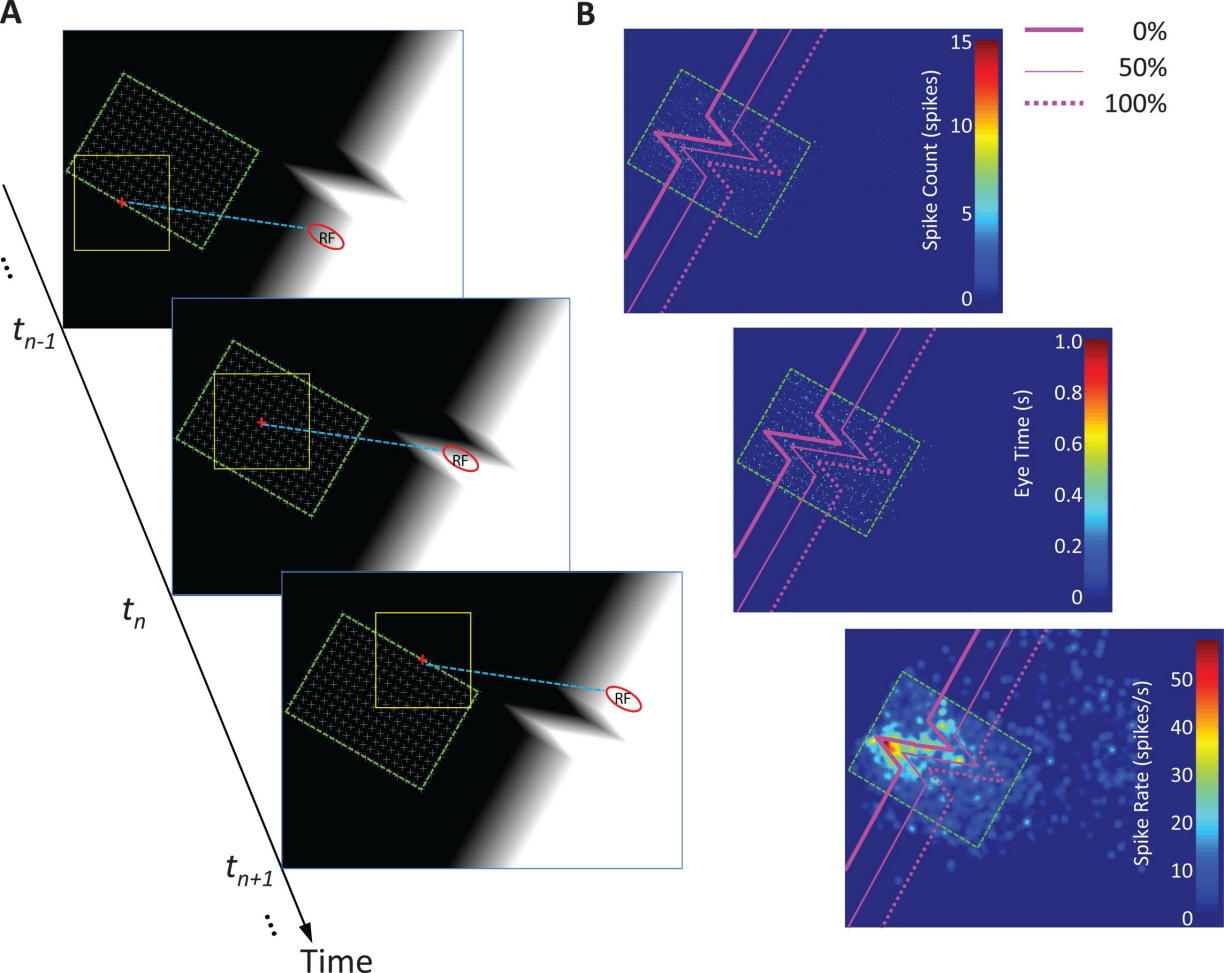
Guided viewing task and the spike rate map. **(A)** Guided viewing. We rotated the visual stimulus to align specific features with the neuron’s preferred orientation (see Materials and Methods for details) in each experimental condition. The green dashed rectangle shows the fixation grid’s boundary. The grid, indicating the candidate positions of the fixation cross, was placed according to the eccentricity of the neuron’s RF. When the monkey fixated his gaze at the grid center, the RF was centered on the visual stimulus (red dots in **Fig 2**). The area of the grid was equal to or slightly larger than each visual stimulus’ ROI. A 2x2 DVA (32 x 32 DU) window (indicated by the yellow square and invisible to the monkey) surrounded the fixation cross (see Materials and Methods for details). When the monkey’s gaze was guided to different fixation cross locations, the RF (indicated by the red oval) covered different regions of the visual stimulus. **(B)** Spike rate map. The green dashed rectangle shows the fixation grid’s boundary (as in (A)). We registered RF positions to different regions of the visual stimulus according to the neuron’s eccentricity and the estimated retina-to-V1 latency. (In other words, we were able to determine what regions of the visual stimulus had stimulated a neuron’s RF at any given time based on that RF’s eccentricity and the estimated latency of the neuron’s responses). Then, we constructed the Spike Count Map by counting the number of spikes that occurred at each image elementary unit (i.e. DU) (top panel). We also constructed the Eye Time Map, i.e. the total time in seconds the eye spent at each image elementary unit (middle panel). Subsequently, we estimated the spike rate map at each image elementary unit by dividing the spike count by the Eye Time. The resulting 2D image was subsequently smoothed with a symmetric 2D Gaussian filter (size of 1 DVA (16 DU) and STD of 0.44 DVA (7DU)) to yield the Spike Rate Map (bottom panel).

Monkeys followed a small fixation cross, which randomly jumped to successive candidate positions within a grid described below). The animals received liquid rewards for keeping their gaze inside an invisible 2x2 DVA (32 × 32 DU) fixation window placed around the cross, for a minimum amount of time (1.20–2.16 s with a mean of 1.77 ± 0.22 s) (**Fig 3A**). Candidate fixation cross positions were constrained to a rectangular (for the Square and Edge and Corners stimuli) or a square (for Star stimulus) grid, which was equal in size to, or slightly larger than, the corresponding ROI (see Visual Stimuli and **Fig 2**for ROI definition). We placed this grid such that when the monkey fixated at its center, the neuron’s RF covered the center of the stimulus (**Fig 2**, red spots). Fixation cross positions were uniformly distributed, and selected with equal probability, within the grid. Specific fixation cross parameters were varied to keep the monkeys at their best performance, as follows. The minimum distance between two cross positions was 0.44–0.88 DVA (7–14 DU) with a mean of 0.65 ± 0.13 DVA; cross size o was 0.50–1.00 DVA with a mean of 0.60 ± 0.06 DVA (10 ± 1 DU); the cross was displayed at each grid position for 2.00–2.85 s with a mean of 2.30 ± 0.19 s. As the monkey followed the cross, the neuron’s RF was located on different parts of the visual stimulus. Neural activity was registered to different parts of the stimulus according to the RF eccentricity and the estimated latency between retina and V1 (set at 60 ms, see “Spike rate and spike rate difference map” and “Conduction latency” sections for details. The minimum number of spikes required to complete each experimental condition was 10,000 (the mean and STD of number of spikes per condition was 35,998 ± 25,981).

### Data analyses

#### Correction of eye signal distortion

To register neural activity to different parts of the visual stimulus using eye position signals, it is crucial to minimize the deviation of eye signals from their true positions. Despite careful system tuning and eye signal calibration, small to moderate distortions in the eye position signals are common, especially for large rotational angles of the eye (i.e. when animals fixate near the monitor’s edges). This is mainly due to a nonlinear effect between the rotational angle and output voltage of the eye coil at large angles, and non-uniformity of the field of drive coils [8]. To null this distortion we took the following three-step approach (**S2 Fig**):

(1) Identification of fixation positions. Recall that the monkey followed a cross and fixated it for a certain time. Since we know the position of the cross and the monkey’s eye position at any given time, we may identify fixation periods in the eye movement signal that are very likely to correspond to the cross positions. We can then use this information to obtain a model to transform the recorded eye positions to the true positions. To do this, we defined a fixation as a blink-free period between two adjacent (micro)saccades; this required defining blinks and (micro)saccades, which we do next. Blinks were defined as periods where the eye velocity (smoothed with a 31-point Boxcar filter) exceeded 3 DVA/s (48 DU/s) for at least 140 ms in a 200 ms period [9]. We also required inter-blink intervals to be at least 50 ms, and thus merged any blinks within 50 ms of each other [9]. After blink removal, we identified (micro)saccades automatically with a modified version of the algorithm developed by Engbert and Kliegl [10–13] with λ = 6 (used to determine the saccadic velocity threshold) and a minimum saccadic duration of 12 ms. Additionally, we imposed a minimum intersaccadic interval of 20 ms so that potential overshoot corrections might not be categorized as new (micro)saccades [14]. To further refine the correspondence between fixation and cross positions, we required: (i) the duration of a fixation, *d*, to be within 500 ms of the average time the cross was displayed for (i.e. T-500≤d≤T+500, where *T* is the average time the cross was displayed in ms) in a session, and (ii) the STD of the spread of eye positions during fixation in both horizontal and vertical directions to be less than 0.5 DVA (8 DU). These criteria make it likely that any given fixation period corresponded to a fixation on the cross.
(2) Registration of fixation positions to cross positions. To match the physical cross positions on the screen with fixation locations (i.e. the actual gaze locations), we needed a transformation that mapped one point set to the other–gaze fixation locations to physical cross positions on the screen. We thus employed the Coherent Point Drift (CPD) algorithm [15]. The CPD algorithm aligns two point sets by solving a probability density estimation problem; one point set is a template in which the points are the centroids of a Gaussian Mixture Model (GMM), and the other point set is the reference to be aligned to. The CPD algorithm fits the GMM centroids to the reference point set by maximizing the likelihood function. Essentially, the CPD algorithm locally forces the centroids to move coherently as a group, which preserves the topological structure of the point set. In our application, the set of fixation locations served as the GMM centroids, and the set of cross locations served as the reference point set. Each gaze fixation location was matched up with a specific cross location. Since the eye signal distortion was moderate, we assumed a non-rigid affine model to correct the error. That is, we assumed that deviations of fixation locations from cross locations could be adequately described by translations, rotations, anisotropic scalings, and skews. After the point set registration, we found the mean and STD of the distance of all the paired points. We deemed a pair of points to be an outlier if the distance between the two points was three STDs away from the mean, and removed the pair from further processing.
(3) Transformation of the eye signals. After we obtained the point pairings between fixation locations and cross locations, we inferred the spatial transformation from the recorded fixation locations to the corresponding cross-locations based on the affine model by using the Image Processing Toolbox of MatLab (MathWorks, Inc.). Once the transform was determined, we applied it to the eye signal to obtain the corrected gaze positions.

#### Spike rate and spike rate difference map

We characterized a neuron’s response to the different visual stimuli with a 2D spike rate map (480 × 640 DUs), which showed the spike rate at each DU (**Fig 3B**). To calculate the spike rate map, we assumed a retina-cortex latency of 60 ms (details in “Conduction latency” section; see also [16,17]) and thus assigned a spike to the monkey’s gaze location 60 ms before the spike time (i.e. if a spike occurred at time *t* ms, we assigned it to the gaze location at *t–* 60 ms). We then obtained the spike rate map by counting the total number of spikes at each DU (spike count map) and divided the total number by the total time the monkey’s RF spent on that DU. For each neuron, we further subtracted the value of baseline activity (described in the “Visual Stimuli” section) from the spike rate map to obtain the spike rate difference. We obtained and aligned the neural activity of spike rate difference in the ROI of each neuron.

#### Conduction latency

The latency of signal conduction from retina to V1 is an important parameter in the present analyses, because its value determines where we register neural activity in the visual stimulus. **S3 Fig** shows the estimated neural responses to corner-folds (at midpoint) for different retina-to-V1 latencies (1, 15, 30, 60, 150, 300, 600, 1200, and 2400 ms). Peak responses occurred around 60 ms across visual stimuli, consistent with previous reports on retina-to-V1 delays (such as the time interval between microsaccade onsets and subsequent bursts of spikes in area V1 (65 ± 46 ms) [16,17]), and thus supporting the appropriateness of our latency choice in the current analyses.

#### Edge RMS (Root-Mean-Square) contrast model

In order to analyze neural responses to Edge at equally spaced contrast points we fit the local RMS contrast with an exponential model $f\left(l\right)=a∙\exp\left(-b∙l\right)+c$, where *l* is luminance level and *a*, *b*, *c* are the parameters to be determined by the fitting procedure. The model was fit with MatLab’s Curve Fitting Toolbox by using the Levenberg-Marquardt algorithm and the LAR method (MathWorks, Inc.).

All relevant data are available from the CRCNS.org public repository: Susana Martinez-Conde, Jorge Otero-Millan, Xoana G. Troncoso, Michael B. McCamy, Stephen L. Macknik (2018); “Single-neuron extracellular recordings from area V1 of awake behaving rhesus monkeys viewing brightness illusions based on Victor Vasarely’s artworks.” http://dx.doi.org/10.6080/K05D8Q1T

## Results

We recorded the responses from 118 single V1 neurons from two rhesus macaques (102 neurons from monkey J and 16 neurons from monkey A), during the presentation of brightness illusions based on Vasarely’s ‘nested squares,’ but discarded 60 neurons before analyzing the data because of technical problems (i.e. noise in the eye coil signal, instability of the recording throughout the session, or poor fixation performance). Thus, we present data from 58 neurons total. Though we started each recording session with the intent to present all three experimental conditions (details below) to each individual neuron, it was challenging to hold the neuronal recordings stable for more than 60–90 minutes at a time. Because data collection usually entailed 30–60 minutes per experimental condition (in addition to the 20–30 minutes necessary to characterize the size, location and orientation of each RF), we were able to test all three conditions in 7 neurons, two conditions in 11 neurons, and a single condition in 40 neurons out of the 58.

Monkeys viewed visual stimuli containing illusory folds–resulting from the alignment of corners of decreasing or increasing luminance—while performing a guided-saccade task (see Material and Methods for details). This required each animal to follow a fixation cross that randomly appeared on one of the nodes of an invisible grid placed over the display, so that the RF of the neuron being recorded was exposed to each region of the stimulus in a uniform manner. The monkey’s eye movements were recorded simultaneously; thus, we were able to register spiking activity to the appropriate image regions and generate a 2D map of neural activity. We presented 3 types of stimuli:

1) Square and Edge (25 neurons), which consisted of concentric (i.e. nested) squares with a luminance gradient decreasing inward to outward, and an edge (the edge of a rectangular pattern comprised of linearly increasing luminance segments, placed on the extended line of one of the diagonals of the square). The Square stimulus was used to measure the neuronal responses to the classical Vasarely illusion [1]. The Edge stimulus was used to compare the neuronal responses to the classical Vasarely illusion to the responses to different contrast levels along a standard edge.
2) Star (30 neurons), which consisted of concentric (i.e. nested) star polygons, each of which was twelve-pointed, equilateral, and equiangular (with luminance decreasing inward to outward). The Star stimulus was used to provide a wide range of illusory corner-fold orientations (to match the preferred orientations of V1 neurons), as well as sharper corner angles than in the Square and Edge condition.
3) Corners (28 neurons), which comprised a spatial sequence (luminance decreasing within the sequence) of pairs of identical isosceles triangles placed in opposite directions and connected successively. The Corners stimulus provided a matching set of dark and bright illusory corner-folds (i.e. same dimensions/properties for the dark/bright corner-folds, only reversing the polarity of the illusion).

In each case, we aligned a particular corner-fold to the preferred orientation of the neuron being recorded. Because we were interested in how corner angles affect neural responses, we quantified the spiking activity at the corner- folds as a function of corner angle. The corner-folds aligned to the neuron’s preferred orientation were S90° for the Square, S52° for the Star, and S40° for the Corners conditions. We also analyzed the neural responses to a non-corner luminance gradient (P180°) in the Square and Edge stimulus, as well as the neural responses along the Edge stimulus.

### Strength of neuronal responses to corner-folds varies parametrically with fold angle

**Fig 4:**
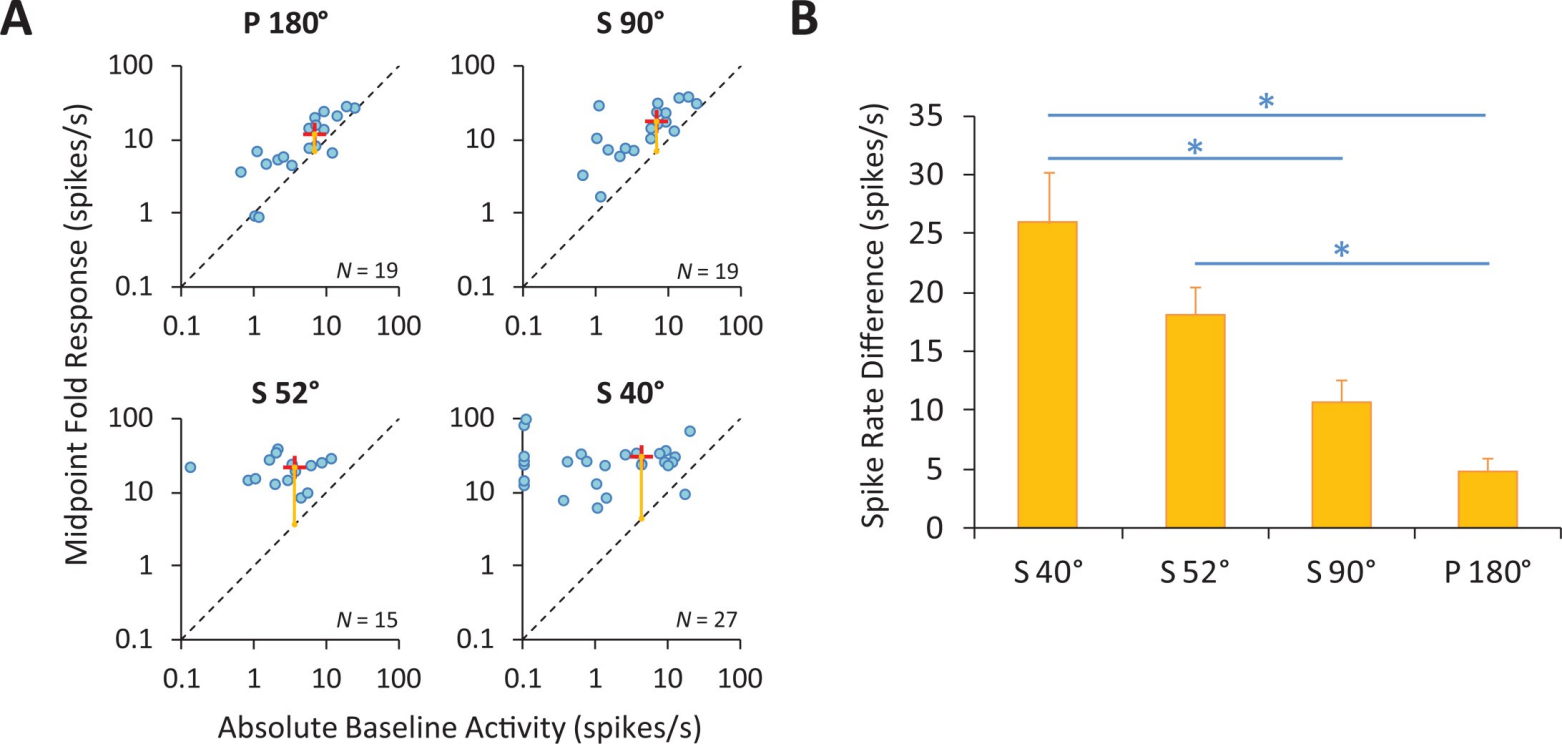
Neural responses to corner-folds depend on corner angle. **(A)** Population data of midpoint corner-fold responses versus absolute baseline activity. Blue points represent responses from individual V1 neurons to corner-folds of three different angles (S90°, S52° and S40°) and to the non-corner gradient (P180°). P180° and S90° were in the Square and Edge stimulus (*N* = 25), S52° was in the Star stimulus (*N* = 30), and S40° was in the Corners stimulus (*N* = 28). The midpoint fold response and absolute baseline activity of each neuron yields the ordinate and abscissa in logarithmic scale of each data point in the scatter plot. The dotted line of unit slope indicates equality between midpoint fold response and absolute baseline activity. The red cross is the mass center of data points. For all folds, most data points and all mass centers fall above the line, indicating a predominance of elevation of midpoint fold response above absolute baseline activity. Furthermore, the length of the yellow vertical line from the mass center to the dotted line of unit slope indicates the average spike rate difference, i.e. the difference: midpoint fold response–absolute baseline activity. The smaller the fold angle, the longer the vertical line, indicating stronger increase of midpoint fold response. **(B)** Spike rate difference for each of the fold responses. As in (A), the average spike rate difference decreases with increasing fold angle. Error bars represent the SEM. * indicates a significant difference (Wilcoxon rank sum test corrected for multiple comparisons with the Benjamini and Hochberg method [18]) between responses (* *p* < 0.05; ** *p* < 0.01; *** *p* < 0.001; see **S1 Table** for exact *p*-values).

We previously showed that the perceived brightness of a corner-fold changes parametrically with corner angle [3,4]. Here, we measured the neuronal responses to equivalent corner-fold stimuli, likewise as a function of corner angle. To draw a proper comparison to our previous psychophysical experiments [3,4], where we measured the perceived brightness at the corner-fold’s midpoint (at 50% luminance), here we first estimated a neuron’s response to a corner-fold by averaging the neural activity in a 0.5 × 0.5 DVA (8 × 8 DU) square region centered on the midpoint of the fold, which we termed the “midpoint fold response” (**Fig 2**). We also calculated the neuron’s baseline activity inside the ROIs, which we called the “absolute baseline activity” (**Fig 2**). Importantly, we found no significant differences in absolute baseline activity across the different visual stimuli presented (**S4 Fig**). Thus, comparisons between different fold angles should reveal differences due to the stimuli, and not just differences between the neurons. **Fig 4A** shows the population data in a scatter plot with midpoint fold response (for P180°, S90°, S52° and S40°) versus absolute baseline activity. The distance (vertical yellow line) from the mass center to the dotted line of unit slope increases as the angle of the fold decreases. This distance indicates the average elevation of neural activity above baseline, which is the difference: midpoint fold response—absolute baseline activity, which we term the “spike rate difference”; note that all neural response analyses, except those in **Fig 4A**, are on the spike rate difference (i.e. response–absolute baseline activity). The distances in **Fig 4B** show the clear trend of diminished spike rate difference with increasing corner angle. The spike rate difference for S40° was significantly larger than that for fold S90° and P180°, and the spike rate difference for S52° was significantly larger than that for P180°. Thus, the sharper the corner angle, the stronger the neural responses.

**Fig 5:**
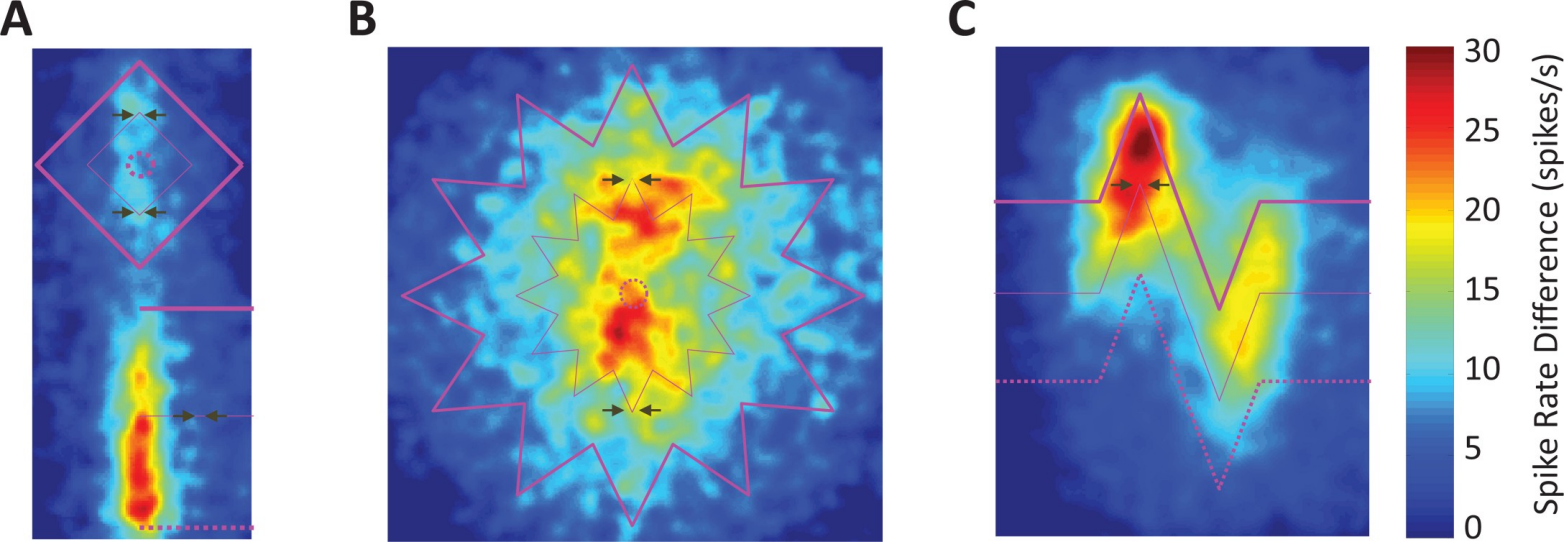
Average spike rate difference maps. The average spike rate differences at each DU in the respective ROIs are shown for **(A)** Square and Edge (*N* = 25), **(B)** Star (*N* = 30), and **(C)** Corners (*N* = 28). The vertical corner-folds are aligned with the preferred orientation of the neurons. The thick, thin, and dashed purple lines indicate the 0%, 50%, and 100% equal luminance lines. Note that in Star and in Square, the 100% equal luminance line contracts to a single point at the center and so we use a small dotted circle to represent this. The paired arrows indicate the midpoints (50% luminance) of corner-folds (i.e. P180°, S90°, S52° and S40°) where midpoint responses were calculated.

For a global view of the data, we further show the 2D spike rate difference map for each visual stimulus (**Fig 5**). The midpoints of corner-folds are emphasized with the paired arrows. The maps also show that neural activity increased with decreasing corner angles. Thus, our results demonstrate that neural responses to corner-folds vary parametrically with corner angle, with smaller (i.e. sharper) angles inducing stronger responses.

### Responses averaged along the entire fold are comparable to those at fold midpoint

**Fig 6:**
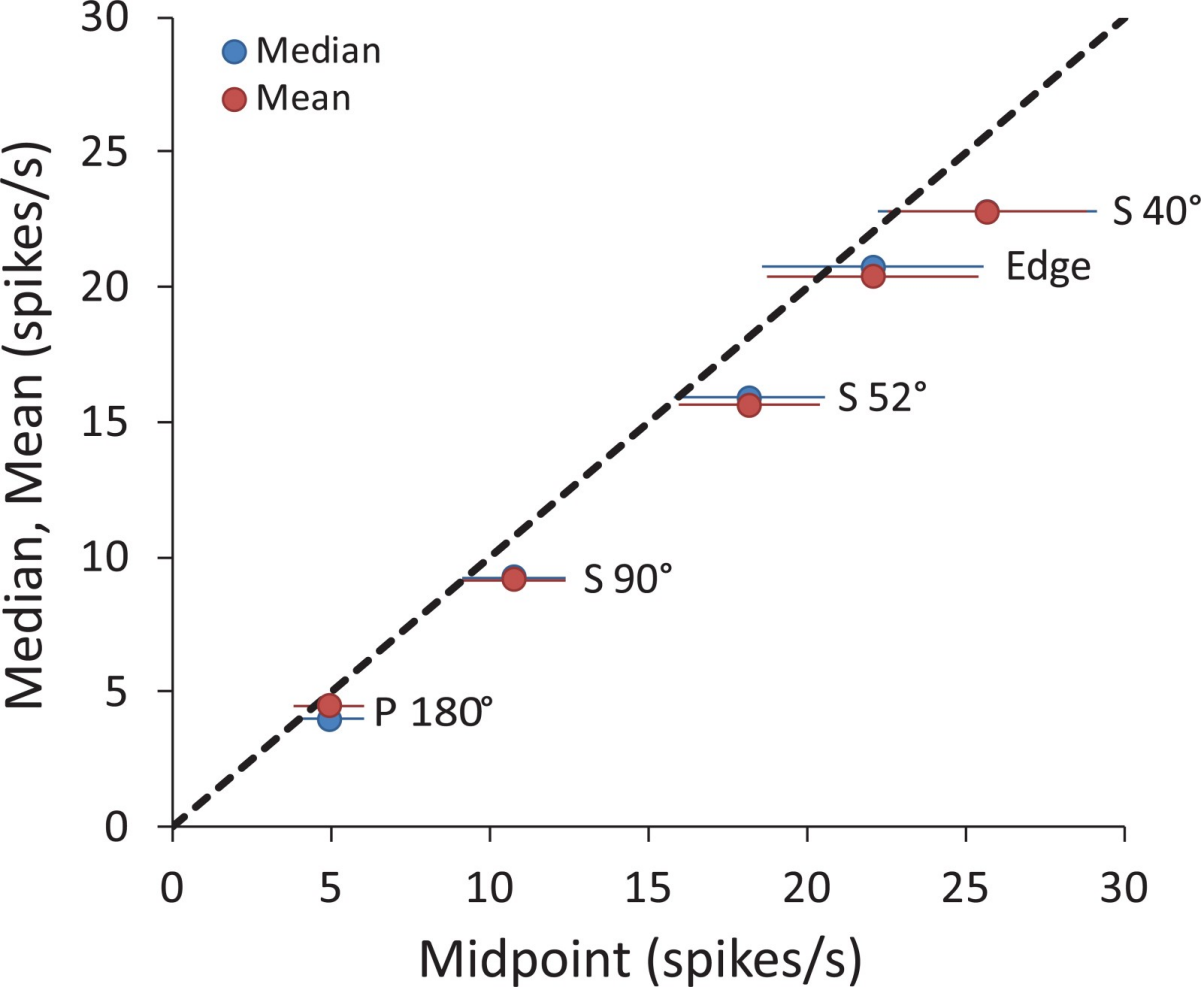
Neuron responses at corner-fold or edge midpoints are equivalent to the mean and median of neuron responses along the corner-folds or Edge. The figure shows the scatter plot of the mean (red dot) and median (blue dot) neuron responses along the folds or Edge versus the neuron responses at fold or Edge midpoints. The dotted line indicates equivalence between the two. Horizontal error bars represent the SEM (the N are as in **Fig 5**).

We first quantified the spike rate difference at the midpoint of the corner-fold, for the purposes of drawing a comparison with our previous psychophysical findings [3,4], where we measured perceived brightness at the corner-fold midpoint (i.e. 50% luminance). Next, we asked whether neural responses at the corner-fold midpoint were representative of neural responses for the entire corner-fold. Thus, we calculated the descriptive statistics of spike rate differences along each corner-fold and Edge. To do this, we segmented a corner-fold spike rate difference map into ten non-overlapping blocks along the luminance gradient, from 0% to 100%. Each block corresponded to a 10% luminance change in height and 0.5 DVA (8 DU) in width. For the Edge, the visual angle subtending 10% luminance change was 0.94 DVA ($\approx \frac{150\text{DU}}{10}\cdot \frac{1\text{DVA}}{16\text{DU}}$); for P180°, S90°, S52° and S40°, it was 0.94, 0.44, 0.94 and 0.63 DVA, respectively, or 15, 7, 15, and 10 DU, respectively. We quantified the mean spike rate difference in each block along the corner-folds and Edge, and then calculated descriptive statistics for these, which are summarized in **Table 1**. We found that the spike rate difference at the corner-fold midpoint was not significantly different from the mean and median spike rate difference along the corner-fold or Edge (Wilcoxon rank sum test, *α* = 0.05; **Fig 6**) and thus was representative of the entire corner-fold response.

**Table 1:** Descriptive statistics of neural responses along edge and corner-folds. The minimum, maximum, mean, and median of the neural activity along the Edge and corner-folds are shown (mean ± SEM), along with the neural activity at the midpoints of the Edge and corner-folds. All values are computed from the spike rate difference map with units of spikes per s.

Stimulus	Corner-fold	Min	Max	Mean	Median	Midpoint
Square & Edge	Edge	7.98±1.60	33.13±5.11	20.44±3.33	20.77±3.48	22.01±3.41
	P 180°	0.18±1.01	10.28±1.53	4.55±1.12	4.03±1.06	4.87±1.15
	S 90°	4.36±1.07	14.61±2.48	9.22±1.61	9.31±1.62	10.69±1.94
Star	S 52°	6.26±1.33	25.39±3.52	15.66±2.23	15.93±2.36	18.10±2.39
Corners	S 40°	9.33±1.69	35.11±4.17	22.81±3.11	22.83±3.48	25.60±4.18

### Neuron responses to corner-folds vs. different contrasts along edge

**Fig 7:**
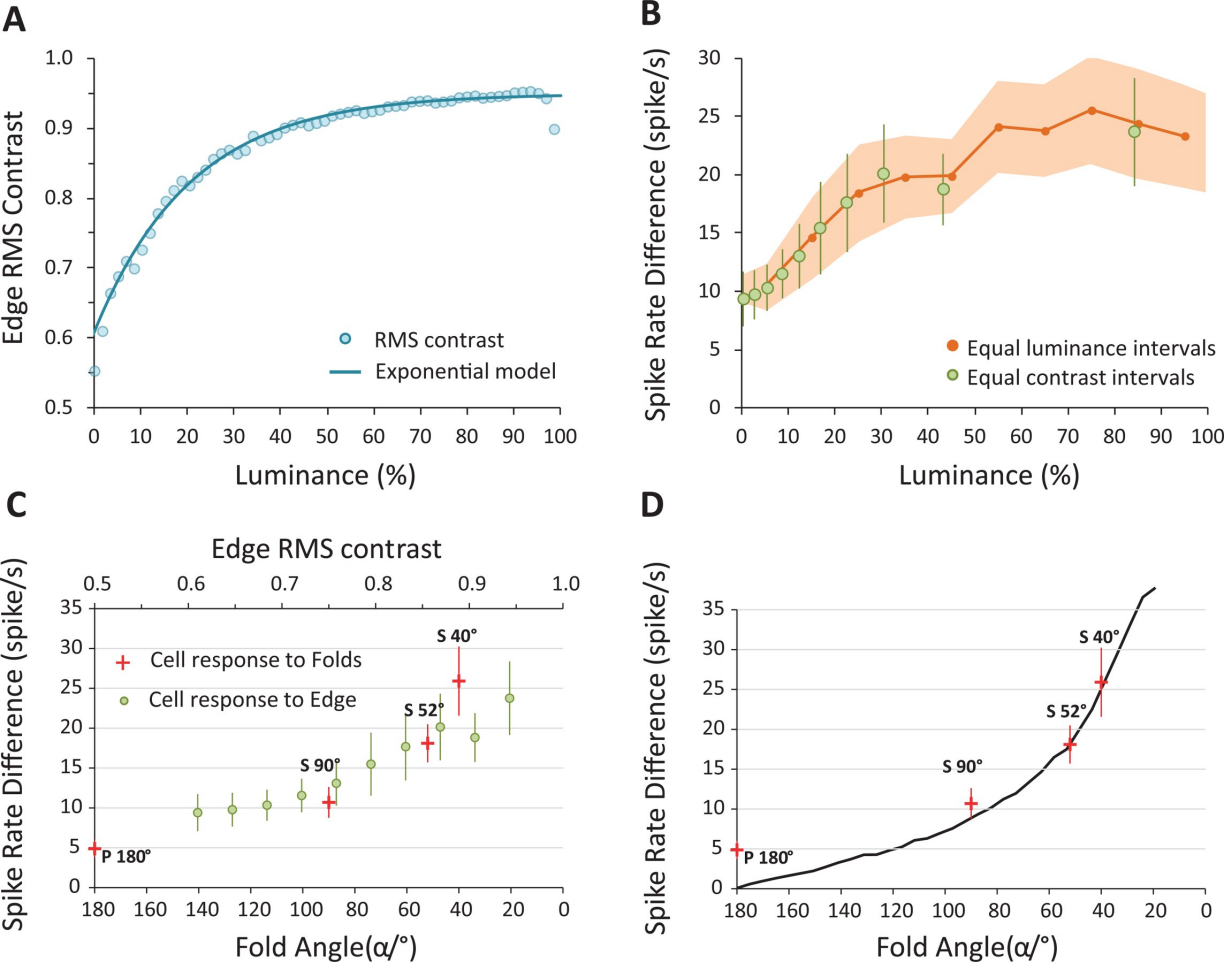
Neural responses to corner-folds vs. those to edge. (**A)** Root-Mean-Square (RMS) contrast along Edge. The light cyan dots show the numerically computed values of RMS contrast at various luminance levels along Edge. The dark cyan line is the exponential fit for Edge RMS contrast: $f\left(l\right)=-0.34\times \exp\left(-0.05∙l\right)+0.95$, where l is the luminance in percentage and $f\left(l\right)$ is the RMS contrast. This model was used to calculate neural responses along Edge at equal RMS contrast intervals. (**B**) Neural responses along Edge. The orange dots indicate the neural responses along the Edge at equal luminance intervals of 10% (red shadows indicate the SEM), while the green dots indicate the neural responses at equal RMS contrast intervals of 0.037. (**C**) Neural responses to corner-folds and Edge. The red crosses represent the average neural response (spike rate difference) to the four corner-fold angles (at fold midpoints). The green dots are the neural response to Edge at equal contrast intervals, as described in (B). (**D**) Neural responses as a function of fold angle (red crosses) compared to DOG output (black line) obtained with similar stimuli [3]. Error bars indicate the SEM (the *N* are as in **Fig 5**).

To quantify the illusory enhancement that occurred at the corner-folds as a function of corner angle, we next matched the neural responses to each of the corner-folds against the neural responses to the different Edge contrast levels. Our goal was to quantify the effect of corner angle on neural activity by determining the contrast level along the Edge that produced a neural response of comparable strength to each of the corner-fold midpoints (which were always 50% in contrast). It is known that cortical neurons display characteristic non-linear responses as a function of luminance contrast, instead of absolute luminance [19,20]. Thus, we examined neural responses as a function of local Root-Mean-Square (RMS) contrast along Edge [21]. Local RMS contrast was computed in a 1 × 1 DVA (16 × 16 DU) square area, centered on the Edge, moving from 0% to 100% luminance in 1.5% luminance steps (**Fig 7A**). This resulted in smooth contrasts ranging from 0.61 to 0.943. In order to acquire neural responses to the Edge at equally spaced RMS contrast intervals, we fit the RMS contrast with an exponential model (*R*^2^ = 0.9983; **Fig 7A**). Then, from 10 equally spaced contrast intervals (from 0.610 to 0.943, sampled at every 0.037 increment) we found the 10 corresponding luminance points along the Edge using the inverse function of the exponential fit. We then calculated the mean neural response centered at these 10 luminance points (in a 1 × 1 DVA (16 × 16 DU) square area) along the Edge. **Fig 7B** shows the neural response computed at equally spaced luminance intervals (red dots) and equally spaced RMS contrast intervals (green dots).

Next, to gauge the strength of the illusory effect as a function of corner angle, we compared the neural responses at each corner-fold midpoint to the Edge responses at different contrasts. Thus, we were able to determine what level of Edge contrast produced a response that was comparable to each of the corner-folds tested. For example, if an 80% Edge contrast produced a neural response that was equivalent to that elicited by a given corner-fold, it would indicate that the corner-fold resulted in a 30% increase in illusory contrast—because the actual contrast of each corner-fold was always 50%, irrespective of its angle.

We found substantial overlap between the range of neural responses to the different corner-folds and the range of neural responses to the Edge contrast levels. **Fig 7C** shows that neural responses to P180° are roughly equivalent to neural responses to a 50% contrast Edge, while neural responses to S40° are close to neural responses to a 90% contrast Edge. In addition, the neural responses to P180° are significantly lower than the responses to a 94% contrast Edge, and the neural responses to S40° are significantly higher than the responses to an Edge of less than 68% contrast (two sample *t*-test corrected; **S2 Table**). These data indicate that increasingly sharp corners produce neural responses equivalent to those generated by substantially higher contrast levels along a straight edge. This neural activity enhancement may be at the heart of the illusory brightness increase experienced by viewers of Vasarely’s nested squares and related illusions.

### Center-surround model vs. V1 neuron responses

Our previous psychophysical studies showed that corner sharpness was linearly related to perceived brightness at the corner-fold midpoint [3,4].

Hurvich originally proposed that Vasarely’s nested squares illusion could be explained by center-surround RFs. That is, he predicted that, in a nested-square pattern, the contrast between the center and the surround regions of concentric RFs would be stronger along corner- than along edge-gradients, resulting in increased perceptual salience at the corners [22] We extended Hurvich's proposal by modeling center-surround RFs as difference-of-Gaussians (DOG) filters [23–25] applying them to corner-fold gradients of varying angles. DOG filter parameters were chosen to match a range of physiological center-surround RFs in the primate LGN ([26–29]; see [3] for details)).

We found that center-surround RFs, modeled as DOG filters, explained the parametric variation in perceptual brightness as a function of corner angle, but did not reproduce the linear relationship found psychophysically [3]. This discrepancy suggested that retinogeniculate (i.e. center-surround) processes might not fully account for our perception of the illusion.

Here we asked whether V1 responses to equivalent visual stimuli were better matched to the psychophysical findings or to the DOG model output. If V1 responses approximated the perceptual data, it would suggest that the illusion could be explained at the level of V1 (or possibly earlier in the visual pathway). In contrast, if V1 responses approximated the output from the DOG model, it would indicate that a complete explanation of the illusion should be found in the extrastriate cortex (i.e. beyond area V1).

**Fig 7D** compares the output from the center-surround model from [3] to the neuronal responses found here from area V1, as a function of corner angle. The two response trends are remarkably similar, suggesting that a) there is little transformation in the neural responses to corner-folds from the retinal/LGN to area V1, and b) the linear relationship previously observed in the psychophysical data likely arises in extrastriate areas of the visual cortex.

## Discussion

In 1961, Barlow proposed that the brain transmits visual data “so that their redundancy is reduced but comparatively little information is lost” [30,31]. This “redundancy-reducing hypothesis” has been used as an explanation of why neurons at the early levels of the visual system (retina/LGN) are suited to perform edge-detection, or contour-extraction. However, redundancy reduction should not be constrained to edges, but should theoretically apply to any feature in the visual scene. Just as edges are a less redundant feature than diffuse light, Attneave proposed in the 1950s that “points of maximum curvature” (i.e. discontinuities in edges, such as curves, angles, and corners—any point at which straight lines are deflected) are also highly informative [32]. If points of high curvature are less redundant than points of low curvature, then sharp corners should also be less redundant than shallow corners. This led to our previous predictions that neural responses to sharp corners should be enhanced compared to those of shallow corners, and thus that sharp corners should be perceptually more salient than shallow corners.

Consistent with this framework, our prior psychophysical studies showed that sharp corners generate illusory folds (i.e. in the nested squares and ABS illusions) that are perceptually more salient than those produced by shallow corners [3,4]. Human fMRI studies of the ABS illusion also supported this conclusion [33].

Here we set out to determine the single-neuron correlates of this phenomenon in the early visual system. To do this, we presented corner-folds of varying angles to awake rhesus monkeys while we recorded from single area V1 neurons whose receptive fields were uniformly exposed to each visual stimulus. Our results showed that V1 neuronal responses varied parametrically with corner angle—with sharp corners generating higher firing rates than shallow ones—consistent with previous human psychophysical and imaging studies [3,4,33]. In contrast with the previous perceptual data, however, we did not find a linear relationship between corner sharpness and the strength of the neural response. Instead, the relationship between corner angle and neural activity was better matched to the DOG output obtained previously with equivalent stimuli [3]. Our combined results suggest there may be little transformation in the neural responses to corner-folds from subcortical to primary visual cortical levels, and that a full neural account of Vasarely’s nested squares and related illusions may only be found in the extrastriate visual cortex.

This possibility is compatible with the known involvement of later cortical stages in the

processing of angles and curves [34,35]. Pasupathy and Connor [35–37] reported selectivity to specific curvature orientations and spatial locations in neurons of area V4, and moreover found that over 70% of V4 neurons tuned to corner angles responded more vigorously to sharp than to shallow angles.

One significant caveat of the present study is the small amount of corner angles that we could test neurophysiologically. Thus, though the observed parametric relationship between neuronal responses and corner angle appears to be non-linear, we cannot be certain that this is indeed the case, given the limited number of angles that we were able to examine.

We also note that recursive signals from V4 have been shown to modulate V1 responses during contour perception [38] and figure-ground segmentation [39]. Thus, the illusions studied here may not be the result of feedforward processing, but could be explained by feedback projections from higher cortical areas—or even by horizontal connections within V1. Though the center-surround RF organization of early visual neurons may be a more parsimonious account of our present data, it does not rule out alternative or complementary cortically-based interpretations.

The present findings offer new insights concerning corner processing in the striate cortex, and will inform future work aimed at determining the stage(s) of the visual pathway in which corner perception may arise.

## Supporting information

S1 Fig Clean visual stimuli. (**A**) Square and Edge. (**B**) Star. (**C**) Corners. All stimuli are drawn to scale.
(EPS)

S2 Fig Correction of eye position signals. (**A**) Distorted eye position signals (black line) and the grid of candidate locations of the fixation cross (red crosses). (**B**) Identified fixation locations before correction. (**C**) Identified fixation locations after correction.
(EPS)

S3 Fig Neural responses to corner-folds as a function of latency. Neural responses at the corner-fold midpoints were estimated as described in the Material and Methods, except that we set the conduction latency from retina-to- V1 from 1 ms to 2400 ms, with the associated neural responses peaking around 60 ms. Error bars indicate the SEM (the N are as in **Fig 5**).
(EPS)

S4 Fig Absolute baseline activity. The distributions of absolute baseline activity in response to three visual stimuli conditions (i.e. Square and Edge, Star, and Corners) are shown in box plots, which indicate the maximum, 25 percentile, median, 75 percentile, and the minimum level of absolute baseline activity in each distribution.
(EPS)

S1 Table Exact *p*-values for Fig 4B, corrected for multiple comparisons (Benjamini and Hochberg method). (DOCX)

S2 Table Comparison of neural responses at different RMS contrasts along the Edge stimulus with the neural responses to the folds at midpoint. Each row shows the mean difference in response at each angle and RMS contrast with its 95% confidence interval, degrees of freedom (DF), t-statistic and p-value (two sample t-test).
(DOCX)

S1 Checklist The ARRIVE guidelines checklist. We followed the ARRIVE (Animal Research: Reporting of In Vivo Experiments) guidelines and the ARRIVE Checklist is available.
(DOC)
