# Supplementary material for: Area V1 responses to illusory corner-folds in Vasarely’s nested squares and the Alternating Brightness Star illusions
Source: PLoS One. 2019 Mar 28;14(3):e0210941. doi: 10.1371/journal.pone.0210941 (PMC6438452; doi:10.1371/journal.pone.0210941)
Supplement: S1 Table — (DOCX) [file pone.0210941.s005.docx]

S1 Table

| Fold 1 | Fold 2 | z-stat | p-value |
| --- | --- | --- | --- |
| 40 | 52 | 0.2998 | 0.2998 |
| 40 | 90 | 0.0013 | 0.0025 |
| 40 | 180 | 0.0000 | 0.0000 |
| 52 | 90 | 0.0192 | 0.0284 |
| 52 | 180 | 0.0000 | 0.0001 |
| 90 | 180 | 0.0237 | 0.0284 |
