# Supplementary material for: Area V1 responses to illusory corner-folds in Vasarely’s nested squares and the Alternating Brightness Star illusions
Source: PLoS One. 2019 Mar 28;14(3):e0210941. doi: 10.1371/journal.pone.0210941 (PMC6438452; doi:10.1371/journal.pone.0210941)
Supplement: S2 Table — Each row shows the mean difference in response at each angle and RMS contrast with its 95% confidence interval, degrees of freedom (DF), t-statistic and p-value (two sample t-test). (DOCX) [file pone.0210941.s006.docx]

S2 Table

| Contrast | FoldAngle | x95_CIL | Diff_Mean | x95_CIU | ndf | t-stat | p-value |
| --- | --- | --- | --- | --- | --- | --- | --- |
| 0.61 | 180° | -10.98 | 4.53 | 20.04 | 24 | 0.57 | 0.333 |
| 0.61 | 90° | -16.8 | -1.29 | 14.23 | 24 | -0.16 | 0.389 |
| 0.61 | 52° | -25.21 | -8.7 | 7.82 | 29 | -1.03 | 0.230 |
| 0.61 | 40° | -30.51 | -16.2 | -1.88 | 27 | -2.22 | 0.038 |
| 0.65 | 180° | -10.61 | 4.9 | 20.41 | 24 | 0.62 | 0.324 |
| 0.65 | 90° | -16.43 | -0.91 | 14.6 | 24 | -0.11 | 0.392 |
| 0.65 | 52° | -24.84 | -8.33 | 8.19 | 29 | -0.99 | 0.241 |
| 0.65 | 40° | -30.14 | -15.82 | -1.51 | 27 | -2.17 | 0.042 |
| 0.68 | 180° | -10.04 | 5.47 | 20.98 | 24 | 0.69 | 0.309 |
| 0.68 | 90° | -15.86 | -0.35 | 15.17 | 24 | -0.04 | 0.394 |
| 0.68 | 52° | -24.27 | -7.76 | 8.75 | 29 | -0.92 | 0.257 |
| 0.68 | 40° | -29.58 | -15.26 | -0.94 | 27 | -2.09 | 0.049 |
| 0.72 | 180° | -8.83 | 6.68 | 22.2 | 24 | 0.84 | 0.274 |
| 0.72 | 90° | -14.64 | 0.87 | 16.38 | 24 | 0.11 | 0.392 |
| 0.72 | 52° | -23.06 | -6.55 | 9.97 | 29 | -0.78 | 0.290 |
| 0.72 | 40° | -28.36 | -14.04 | 0.27 | 27 | -1.92 | 0.066 |
| 0.76 | 180° | -7.3 | 8.21 | 23.73 | 24 | 1.04 | 0.228 |
| 0.76 | 90° | -13.11 | 2.4 | 17.91 | 24 | 0.30 | 0.376 |
| 0.76 | 52° | -21.53 | -5.01 | 11.5 | 29 | -0.59 | 0.330 |
| 0.76 | 40° | -26.83 | -12.51 | 1.8 | 27 | -1.71 | 0.093 |
| 0.8 | 180° | -4.9 | 10.61 | 26.13 | 24 | 1.34 | 0.160 |
| 0.8 | 90° | -10.71 | 4.8 | 20.31 | 24 | 0.61 | 0.326 |
| 0.8 | 52° | -19.13 | -2.61 | 13.9 | 29 | -0.31 | 0.376 |
| 0.8 | 40° | -24.43 | -10.11 | 4.21 | 27 | -1.38 | 0.151 |
| 0.83 | 180° | -2.71 | 12.81 | 28.32 | 24 | 1.62 | 0.108 |
| 0.83 | 90° | -8.52 | 6.99 | 22.5 | 24 | 0.88 | 0.265 |
| 0.83 | 52° | -16.94 | -0.42 | 16.09 | 29 | -0.05 | 0.395 |
| 0.83 | 40° | -22.24 | -7.92 | 6.4 | 27 | -1.08 | 0.218 |
| 0.87 | 180° | -0.23 | 15.28 | 30.79 | 24 | 1.93 | 0.065 |
| 0.87 | 90° | -6.05 | 9.46 | 24.97 | 24 | 1.20 | 0.192 |
| 0.87 | 52° | -14.47 | 2.05 | 18.56 | 29 | 0.24 | 0.384 |
| 0.87 | 40° | -19.77 | -5.45 | 8.87 | 27 | -0.75 | 0.297 |
| 0.91 | 180° | -1.56 | 13.95 | 29.46 | 24 | 1.76 | 0.086 |
| 0.91 | 90° | -7.38 | 8.14 | 23.65 | 24 | 1.03 | 0.230 |
| 0.91 | 52° | -15.79 | 0.72 | 17.24 | 29 | 0.09 | 0.394 |
| 0.91 | 40° | -21.09 | -6.78 | 7.54 | 27 | -0.93 | 0.255 |
| 0.94 | 180° | 3.37 | 18.89 | 34.4 | 24 | 2.39 | 0.028 |
| 0.94 | 90° | -2.44 | 13.07 | 28.58 | 24 | 1.65 | 0.103 |
| 0.94 | 52° | -10.86 | 5.66 | 22.17 | 29 | 0.67 | 0.314 |
| 0.94 | 40° | -16.16 | -1.84 | 12.48 | 27 | -0.25 | 0.382 |
